# Nanobody-Based Delivery Systems for Diagnosis and Targeted Tumor Therapy

**DOI:** 10.3389/fimmu.2017.01442

**Published:** 2017-11-02

**Authors:** Yaozhong Hu, Changxiao Liu, Serge Muyldermans

**Affiliations:** ^1^Department of Pharmaceutical Engineering, School of Chemical Engineering and Technology, Tianjin University, Tianjin, China; ^2^Laboratory of Cellular and Molecular Immunology, Vrije Universiteit Brussel, Brussels, Belgium; ^3^State Key Laboratory of Drug Delivery Technology and Pharmacokinetics, Tianjin Institute of Pharmaceutical Research, Tianjin, China

**Keywords:** nanobody, targeted cancer therapy, drug delivery, intracellular targeting, type III secretion system, molecular imaging

## Abstract

The development of innovative targeted therapeutic approaches are expected to surpass the efficacy of current forms of treatments and cause less damage to healthy cells surrounding the tumor site. Since the first development of targeting agents from hybridoma’s, monoclonal antibodies (mAbs) have been employed to inhibit tumor growth and proliferation directly or to deliver effector molecules to tumor cells. However, the full potential of such a delivery strategy is hampered by the size of mAbs, which will obstruct the targeted delivery system to access the tumor tissue. By serendipity, a new kind of functional homodimeric antibody format was discovered in camelidae, known as heavy-chain antibodies (HCAbs). The cloning of the variable domain of HCAbs produces an attractive minimal-sized alternative for mAbs, referred to as VHH or nanobodies (Nbs). Apart from their dimensions in the single digit nanometer range, the unique characteristics of Nbs combine a high stability and solubility, low immunogenicity and excellent affinity and specificity against all possible targets including tumor markers. This stimulated the development of tumor-targeted therapeutic strategies. Some autonomous Nbs have been shown to act as antagonistic drugs, but more importantly, the targeting capacity of Nbs has been exploited to create drug delivery systems. Obviously, Nb-based targeted cancer therapy is mainly focused toward extracellular tumor markers, since the membrane barrier prevents antibodies to reach the most promising intracellular tumor markers. Potential strategies, such as lentiviral vectors and bacterial type 3 secretion system, are proposed to deliver target-specific Nbs into tumor cells and to block tumor markers intracellularly. Simultaneously, Nbs have also been employed for *in vivo* molecular imaging to diagnose diseased tissues and to monitor the treatment effects. Here, we review the state of the art and focus on recent developments with Nbs as targeting moieties for drug delivery systems in cancer therapy and cancer imaging.

## Introduction

**Figure 1:**
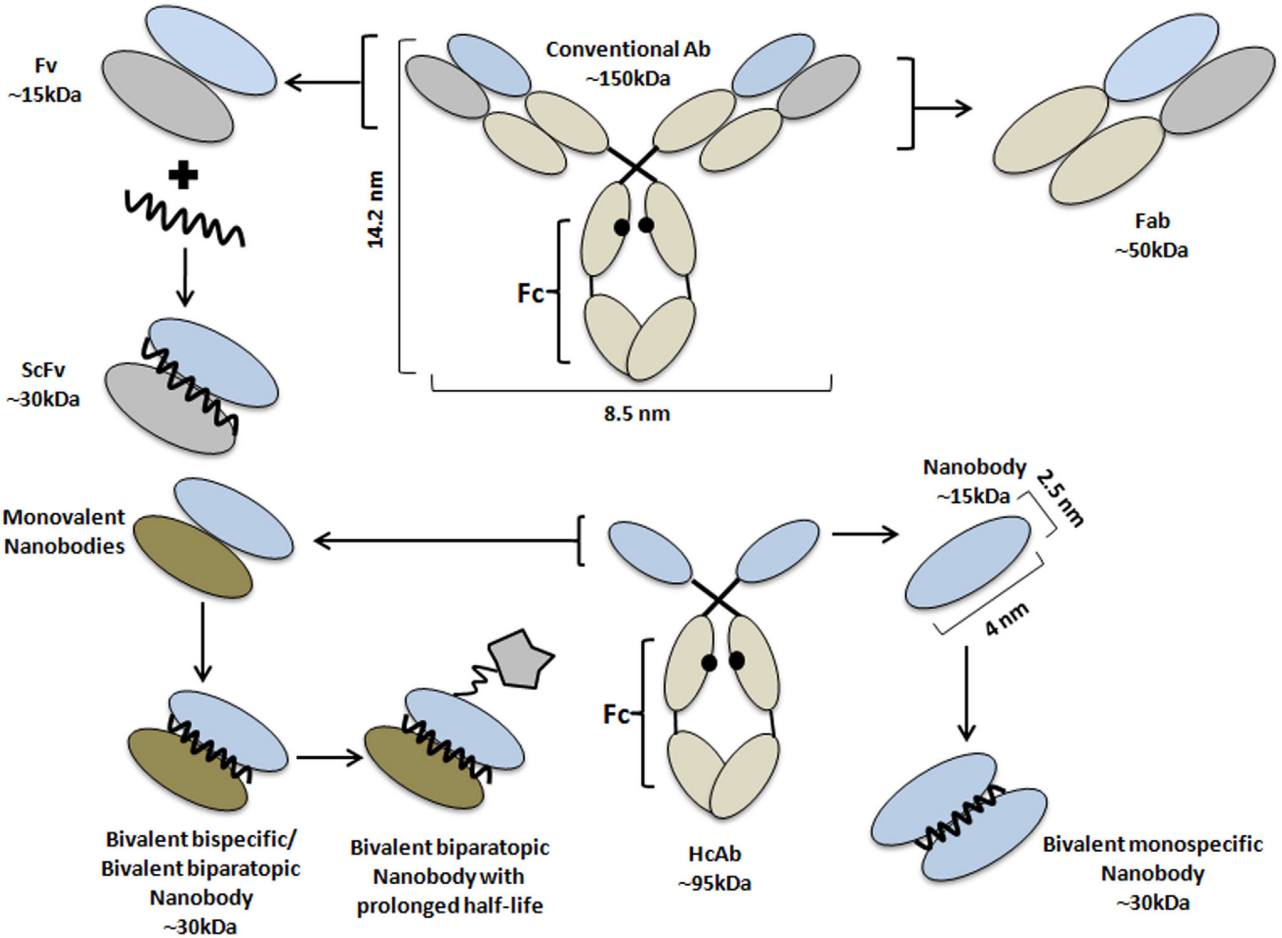
Schematic representation of antibodies and their derivatives from conventional and heavy chain-only antibodies. Schematic structure of a monoclonal antibody (central top part) and its derivatives: Fab (right, top), Fv, and scFv (left, top part); and of a HCAb (central, lower part), together with its antigen-binding fragment, known as VHH or nanobody (Nb) (right, lower part). Besides the monovalent format, Nbs have been engineered into bivalent monospecific constructs (lower part, right). Two different Nbs can be fused into (i) a biparatopic construct where each Nb recognizes a different epitope on the same molecule or (ii) a bispecific construct targeting two independent molecules (lower, left part). The fusion of the Nb-based construct with a large molecule (star-like shaped) or with an Nb with specificity for albumin are standard strategies to prolong the half-life of the construct in the bloodstream. The molecular weight of each Ab format is also given.

To date, the development of effective strategies for cancer therapy remains a huge challenge. The conventional chemotherapy and radiotherapy appear to have a potent effect to kill tumor cells, but they also eliminate healthy cells. Therefore, massive attention went to the development of more effective curable options by targeted cancer therapy (1). Over the years, antibodies have been employed, first as antivenom therapeutic and later as a valued research and clinical diagnostic tool. The first injection of monoclonal antibodies (mAbs) into patients dates back some 30 years ago (2, 3). But murine mAbs elicit immunogenicity problems in patients. Nevertheless, mAb-based cancer therapy has obtained remarkable successes, emphasizing the attention to evolve therapeutic treatments into a personalized curable proposal. To date, the Food and Drug Administration has approved over 30 mAbs for clinical application. Among these mAbs, seven blockbusters are combatting tumors, including Rituximab (anti-CD20), Trastuzumab (directed to HER2), Bevacizumab (anti-VEGF), Alemtuzumab (anti-CD52), Cetuximab, Panitumumab, and Matuzumab (all targeting EGFR) (4, 5). These mAbs were selected for their capacity to disturb the normal function of their targets in tumor cells. The intact mAbs, containing a fully functional Fc domain, evoke antibody-dependent cell-mediated cytotoxicity (ADCC). In addition, mAbs showed significant potential for tumor diagnosis through molecular imaging (6, 7). The mAbs have also been engineered to carry various toxic loads to produce immunotoxins, antibody drug conjugates (ADCs), nanomedicines, or nanoparticles (NPs) encapsulating cytotoxic agents, that work as drug delivery systems (8). For example, mAbs have been directly conjugated to cytotoxic drugs [e.g., auristatin, maytansine, calicheamicin, or doxorubicin (DOX)] and several of these ADCs reached the clinical trials (9). Nevertheless, the large size of mAb (MW 150,000; dimensions: 14.2 nm × 8.5 nm × 3.8 nm), which might be further increased after conjugation with NPs, constitutes a manifest drawback. An enlarged size will lead to a suboptimal biodistribution and a limited tumor penetration (10). Considerable efforts have been put into the development of smaller antibody formats (11, 12), such as the naturally derived or recombinant antigen-binding fragment (Fab; ~50 kDa), variable fragment (Fv; ~28 kDa), or single-chain variable fragment (scFv; ~30 kDa) (Figure 1) (13). To reinstall the bivalency and concomitant avidity effects, the minibody (an engineered antibody fragment made by fusing the scFv binding domain to human CH3) was introduced as a better candidate (14). Important successes were obtained with these size-reduced Abs of which some reached clinical trials, however, detailed immunogenicity studies are underrepresented and research is still ongoing (15–17).

By serendipity, a new type of antibody naturally devoid of light chains and their first constant domain (CH1) in the heavy chain was discovered in the early 90s in camelids (18). These heavy chain-only antibodies (HCAbs) exhibit a similar affinity to their cognate antigen compared to conventional antibodies, despite only one single variable domain (VHH) is involved in antigen recognition (19). It was demonstrated that the autonomous VHH retains its full antigen-binding potential, and it was considered to be the smallest natural intact antigen-binding fragment (20, 21). With a size of below 15 kDa and dimensions in the nanometer range (~2.5 nm in diameter and ~4 nm in height), the VHH molecule was also named nanobody (Nb, Figure 1). The immense efforts in the Nb research field underscoring the remarkable prospects of these molecules formed eventually the basis for the foundation of spin-offs such as Ablynx, Chromotek, Agrosavfe, QvQ, Camel-IDS, Hybrigenics, Confo-Therapeutics, and many other companies offering the technology of generating and selecting Nbs (20, 22). The focus of all these companies ranges from service providers to developing therapeutic Nbs, currently tested in clinical trials (23) with ~9 candidates in advanced stage and more than 15 at the discovery and preclinical stage.

In this report, the beneficial characteristics of Nbs will be reviewed and different Nb conjugation systems for tumor targeting and drug delivery will be discussed, as well as strategies to target intracellular tumor markers. The latter will not only facilitate the exploration of new potential therapeutic approaches but also expand our understanding of particular signaling cascades. Finally, the *in vivo* molecular imaging using Nbs will be summarized.

## Characteristics of Nbs

The ontogeny and emergence of dedicated genes to produce HCAbs in camelids, including VHH domains generated after gene rearrangement events have been comprehensively covered (22, 24–27).

### Nbs Are Easily Selected by Phage Display

The VHH repertoire from peripheral blood cells of the immunized camelid is cloned and phage displayed to retrieve Nbs with highest affinity and specificity for the target (28). The procedure has been adapted to construct large non-immune (naive) or synthetic Nb libraries, from which to select binders. Naive libraries use the VHH repertoire of non-immunized animals. For synthetic libraries, the codons of the antigen-binding loop regions of a robust VHH scaffold are randomized. In all cases, selected Nbs can be produced easily in microorganisms, mammalian cells, or plants (29–32).

### The Smaller Size of Nbs Assists in Reaching and Recognizing Unique Epitopes

The Nb holds great promises (33), mainly due to a unique paratope architecture, monomeric, and robust behavior (34–36) and favorable solubility (21). Due to their small size, a rapid extravasation of intravenously administered Nbs and diffusion into tissues is obtained to deliver interesting reagents to the target. Many Nbs possess a long complementarity determining region 3 (CDR3), forming a finger-like structure that penetrates into cavities on the antigen surface (36). For those VHHs that do not have a long CDR3, the prolate shape of the Nb creates a convex paratope that interacts deeply into antigen concave surfaces. Consequently, Nbs are directed against unique antigen epitopes that are low or not antigenic for classical antibodies (37–39).

### The Smaller Size of Nbs Is Beneficial for Engineering

The small size and monomeric single-domain nature forms the basis for the flexible engineering of Nbs. Engineering of Nbs facilitates the conjugation of additional proteins, reporter molecules, or drugs. Most methods, employed for the chemical conjugation, depend on presence of lysines. However, the occurrence of multiple lysines (on average 3–4 per Nb) and their random conjugation creates a mixture of conjugates whereby a fraction might have lost its antigen-binding capacity when lysines within the antigen-binding region reacted. The introduction of an extra cysteine at a distant location from the paratope and preferably at the C-terminal end of the domain remediates these issues (40, 41). Alternatively, the C-terminal end of the Nbs have been equipped with short peptide tags, such as the Sortag that undergoes the Sortase A-mediated protein ligation reaction to attach any probe (42, 43).

### Inconveniences of Nbs and How to Remediate

The minimal size of an Nb is often considered as an advantage; however, it might also be a handicap. For example, all molecules with a size below 50,000 Da are rapidly cleared from the bloodstream through kidney glomerular filtration. Although a fast blood clearance of Nbs is certainly beneficial for non-invasive *in vivo* imaging (33, 44, 45), for optimal tumor therapy, a longer blood residence time would permit lower injected doses, longer time intervals between two consecutive administrations and still yield a higher load of Nb-based drug at the target. To increase the blood residence time, Tijink et al. (46) generated a tandem fusion of a bivalent Nb against EGFR with an Nb cross-reacting with mouse and human serum albumin (α-EGFR-αEGFR-αAlb, Figure 1) (47). Since human serum albumin has a half-life of around 19 days (48), the half-life of the bispecific trivalent α-EGFR-αEGFR-αAlb was prolonged to around 2–3 days in mice. Furthermore, compared to the monovalent Nb, the longer circulation of the trivalent Nb in blood increased its tumor uptake as well. Similar levels of tumor loading were noted with the trivalent Nb and Cetuximab, while a faster and deeper tumor penetration was obtained with the former (47).

For conventional antibodies, it is well established that upon antigen binding, the ADCC and complement-dependent cytotoxicity are triggered by the Fc region (47, 49, 50). These two mechanisms are known to be important in the process of tumor eradication, as they both contribute to activation of cell lysis, and hence apoptosis cascades (47). For this reason, it was proposed to extent Nbs with an Fc region, although the advantages of a small size (extravasation, tumor penetration) will be lost (50).

### High Stability of Nbs Admit Their Application under Stringent Conditions

Nanobodies seem to be extraordinarily resistant when exposed to various stress conditions. The Nbs have a long shelf-life and tolerate storage for several months at 4°C, and even longer at −20°C, while maintaining full antigen-binding capacity. Incubating Nbs at 37°C for several weeks seems to be well tolerated as well (28). Although some reports indicate that Nbs might resist temperatures above 90°C (35), this will be more an exception than the rule and Nbs are certainly not always refolding quantitatively upon heat denaturation. Also exposure to elevated pressure does not seem to harm the Nbs. Altogether, most Nbs exhibit a high stability against elevated temperature, high pressure, or chemical denaturants as demonstrated by thermo fluorescence or circular dichroism measurements (34, 51, 52).

### Low Immunogenicity of Nbs

The detailed Nb sequence information (21) revealed that VHHs share a high degree of sequence identity with human VHs (of family 3). This feature is considered to contribute to the low immunogenicity, thus allowing prolonged and repeated administrations of Nbs in patients (53). Furthermore, strategies have been developed to humanize Nbs to minimize the possible immune reaction of patients (54, 55). Data from Phase I clinical trials performed by Ablynx (Belgium) also support the notion that Nbs are endowed with low immunogenicity (56, 57).

As it is difficult for Nbs, as well as for other proteins, to migrate across cell membranes, most current investigations had a focus on extracellular targets, such as receptor ligands or transmembrane proteins. However, possible applications of Nbs directed against intracellular tumor markers have been proposed. For example, scFv or Nbs might be transcribed and translated inside the tumor cell. Such intracellular antibodies (known as intrabodies), when folded properly might immediately target the intracellular antigen protein. Groot et al. (58, 59) produced intrabodies against HIF-1α and evaluated its targeting efficacy. Obviously, explorative experiments with Nb-based tools either expressed intracellularly (intrabodies) or introduced *via* viral vectors are underway (60–62).

## Nb-Conjugated Particles for Therapy and Diagnosis

From the very beginning, the potential of Nbs as cancer therapeutic agent has been evaluated, whereby the Nb targets the ectodomain or cell surface exposed loops of receptors or biomarkers, aiming at the inactivation of the transcriptional pathways or signaling cascades. In the following section, the therapeutic agents and the different formats of drug delivery systems based on Nbs will be described.

### Nbs with an Intrinsic Therapeutic Activity

To date, the most investigated extracellular targets for Nbs include EGFR1 or EGFR2 (HER1 and HER2, respectively), vascular endothelial growth factor receptor-2 (VEGFR2), c-Met and CXCR7, or hepatocyte growth factor (HCG), which all play a crucial role in making a link with the signaling cascades. Binding of Nbs to these tumor markers can potentially block the signaling pathways to halt the growth and proliferation of tumor cells. As such, Nbs against EGFR and c-Met have been evaluated (47, 63). Both Nbs showed potent antagonistic effects *in vitro*, as well as an inhibition of the tumor growth *in vivo* in case of a trivalent biparatopic anti-EGFR Nb 7D12-9G8-Alb (47).

Furthermore, Nbs have been developed to combat different infections and diseases, such as thrombotic thrombocytopenic purpura (64, 65), respiratory syncytial virus (66), and rheumatoid arthritis (67–69). These Nbs reached various stages of preclinical or clinical testings. Some particular Nbs are being developed as allosteric inhibitors that are able to modulate the enzymatic activity of their target protein, such as carbonic anhydrase (CAIX) (70), which plays a significant role for hypoxic tumor cells so that the enzymatic CAIX neutralization with Nbs could reduce malignancy and survival of tumor cells. All these results will expand the research focus and stimulate applications of Nbs for cancer diagnosis and therapy. Theoretically, potent and intrinsic effective Nbs that can completely inhibit tumor growth and lead to cell death should be employed, rather than Nbs that are just inhibiting tumor cell proliferation.

### Nb-Toxin Conjugation

However, most Nbs do not exhibit an inherent therapeutic activity, but need to be conjugated with a toxic load or any other effector function. In these applications, the conjugated Nbs are employed for drug delivery, irrespectively whether the conjugate is a single effector domain or a nanocargo containing antitumor drugs (71–73).

The conjugation of Nbs with an enzyme or toxin molecule increases the Nb circulation time in blood due to its enlarged size. Therefore, the constructs become more effective to transfer their load to tumors or diseased tissues. Two strategies can be applied for conjugation, either by chemical conjugation or by gene fusion of the Nb and a toxic protein and cloning in an expression vector (55, 74). For chemical conjugation, the conjugation of the effector moiety to the Nb—mostly to lysine residues—might be heterogeneous as several lysines are present in the Nb and if a Lys in the CDR reacted then the reactant might shield the CDRs from access to antigen, thus resulting in a decrease or loss of antigen recognition by Nbs. In another strategy, a single cysteine—inserted at the C-terminal end of the Nb—allows for a unique site-directed conjugation of a toxic load distant to the paratope so that the disturbance of antigen binding will be minimal (41). Recently, the Sortase A-mediated modification of the C-terminal end of Nbs has been explored to attach chelators and nuclides (42, 43). Anyway, the antigen-binding properties must be confirmed after conjugation. Hence, it is necessary to ensure the accumulation and retention of specific Nbs at the tumor tissue, rather than normal healthy tissue.

#### Anti-Carcinoembryonic Nbs with *Enterobacter cloacae* β-Lactamase

In 2004, Cortez-Retamozo et al. (75) introduced the genetic conjugation of anti-carcinoembryonic Nbs to *E. cloacae* β-lactamase, which is an enzyme with excellent catalytic properties for converting a non- or low-toxic prodrug into a potent cytotoxic agent. *In vivo* biodistribution and therapeutic activity of the conjugation was evaluated in nude mice bearing LS174T xenografts. Effective accumulation of the Nb conjugate at the tumor xenograft was noticed and no, or very low, accumulation in kidneys. Regression of the grafted tumor was observed in mice and even complete remission was obtained in this antibody-directed enzyme prodrug therapy model. Although the bacterial origin of β-lactamase will make the immuno-enzyme highly immunogenic and therefore less practical, the study provides the proof of concept to generate highly cytotoxic compounds in the vicinity of the tumor and shows its potential as a promising approach for cancer therapy.

#### Anti-EGFR Nbs with Tumor Necrosis Factor-Related Apoptosis-Inducing Ligand (TRAIL)

In 2012, a unique conjugation based on anti-EGFR Nbs was introduced for malignant glioblastoma multiforme (GBM). As demonstrated, both neural stem cells (NSCs) and mesenchymal stem cells can migrate toward brain tumors. In this study, bivalent and bispecific Nbs against EGFR were conjugated to TRAIL and packaged into lentivirus (LV) virions to transduce NCS. The secretion of specific Nbs and Nb-TRAIL from engineered NSCs was confirmed and NSCs retained the ability to differentiate. Furthermore, these Nb constructs secreted from NSCs were designed to target GBM tumor tissues, which show an enhanced EGFR expression (76). The therapeutic effect of anti-EGFR Nbs and their variants was evaluated both *in vitro* and in xenografted models. NSC released anti-EGFR Nbs can inhibit EGFR signaling dramatically *in vitro* and reduced the tumor growth in mice bearing GBM. By taking advantage of tropism of the NSCs that could provide on-site delivery of therapeutic Nbs, significant inhibitory effects on GBM were noticed. Direct comparison of the inhibition activity between the bivalent anti-EGFR Nb and the Nb-TRAIL conjugate further revealed that the combined therapeutic approaches were more potent (76). The NSC-delivered Nbs inhibit the proliferation and migration, whereas their conjugation with cytotoxic molecules enhances the therapeutic efficacy, significantly.

#### Anti-VEGFR2 Nbs with Recombinant *Pseudomonas* Exotoxin A

The conjugation of anti-VEGFR2 Nbs with recombinant *Pseudomonas* exotoxin A (PE38) was proposed to inhibit growth of tumors, highly expressing VEGFR2 (77). PE38 was designed to enter the cell in an endocytic vesicle and to bind to the ADP-ribosylating elongation factor II to kill subsequently tumor cells. As demonstrated by an *in vitro* proliferation assay, this conjugation system could efficiently recognize VEGFR2 expressed on the surface of 293KDR cells and inhibit their proliferation *in vitro* (77). Thus, this anti-VEGFR2–PE38 conjugate act as a potent immuno-cytotoxic effector; however, data of an *in vivo* evaluation have not been reported.

In conclusion, the conjugation of Nbs and toxins combines the advantages of the tumor-specific targeting Nbs and the tumor killing toxins within one molecule. In this case, an effective cell penetration was also achieved, which will help to enhance the therapeutic efficacy *via* this particular effector domain. Several Nb conjugates are in the pipeline for research purposes or clinical evaluation.

### Nbs As Targeting Modules for Drug Delivery Systems

**Figure 2:**
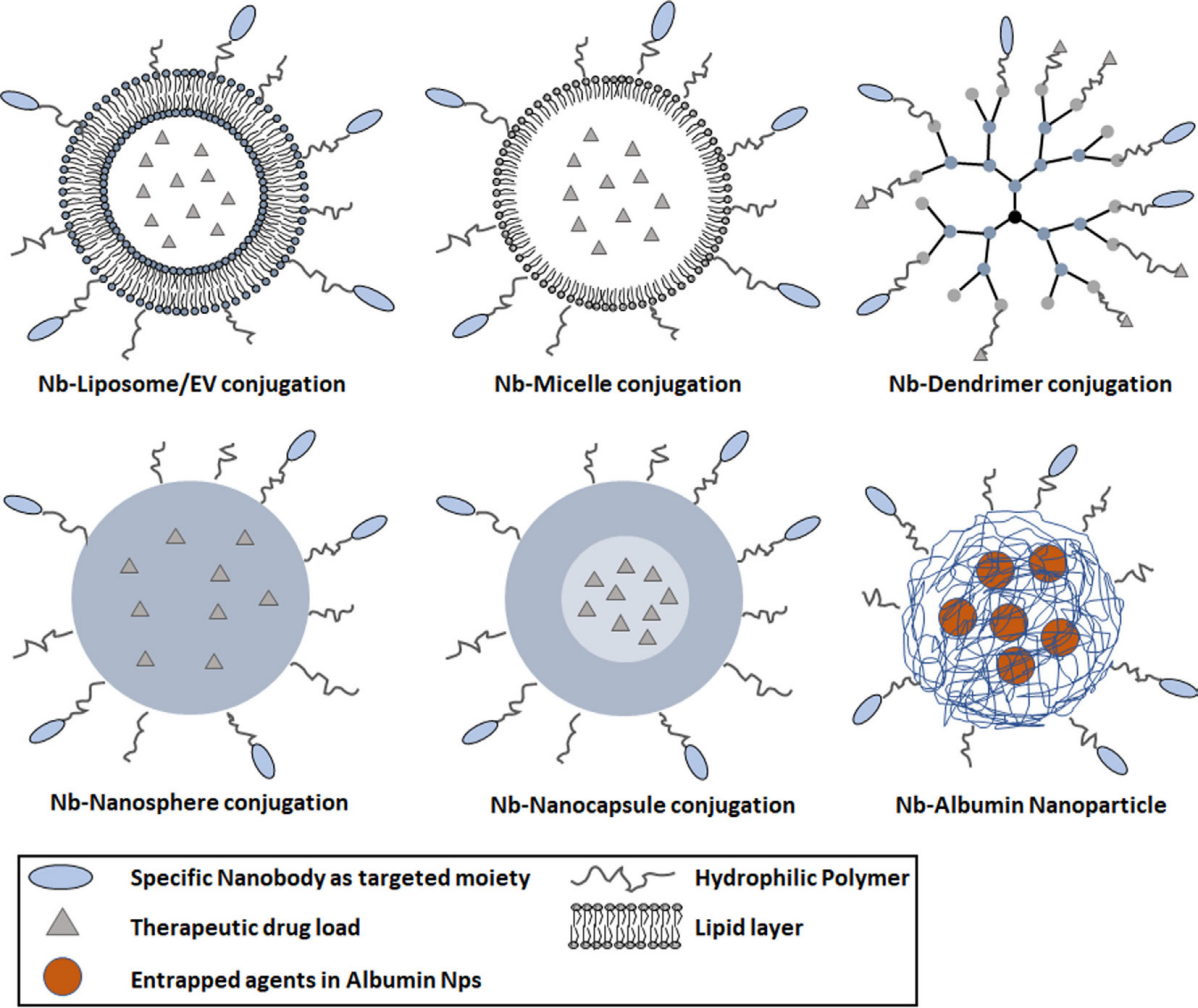
Schematic diagram representing various types of nanoparticles (NPs) decorated with nanobodies (Nbs) for targeted cancer therapy. Commonly used NPs comprise various materials, such as liposomes (100–400 nm), micelles (10–100 nm), dendrimers (3–20 nm), nanospheres (1–100 nm), and nanocapsules (10–1,000 nm). The blue parts of the polymer NP represent the solid hydrophobic polymer matrix with optionally an aqueous core. The nanosphere is composed of a solid polymer matrix, able to encapsulate hydrophobic drugs. The nanocapsule is composed of a spherical polymeric matrix with an aqueous or oily core (light blue part in lower right panel). The poly-ethylene glycol-ylation prolongs the circulation of NPs in the bloodstream; antigen-specific Nbs are conjugated to the surface of NPs for targeting purposes.

Drug delivery systems also involve nano-sized drug carriers or NPs with a diameter below 200 nm. The design of nanoscale vehicles for drug delivery has been one of the most exiting strategies in medicine and pharmaceutical technology. Different drug delivery systems based on NPs have been developed, including inorganic, magnetic, and polymeric NPs (Figure 2) (78). These systems can protect drugs against oxido-reduction and enzymatic reactions, increase their bioavailability by reducing the effective dose and they will diminish the potential immunogenicity of the drug. The packaged, administered toxic compounds can avoid damage and negative side effects to normal tissues, solubilize hydrophobic drugs in lipidic bilayers (e.g., liposomes) or hydrophobic cores (micelles). The NPs allow administering larger amounts of drugs in one single dose, and the slow but prolonged drug release will result in a reduced frequency of the administration (79).

**Figure 3:**
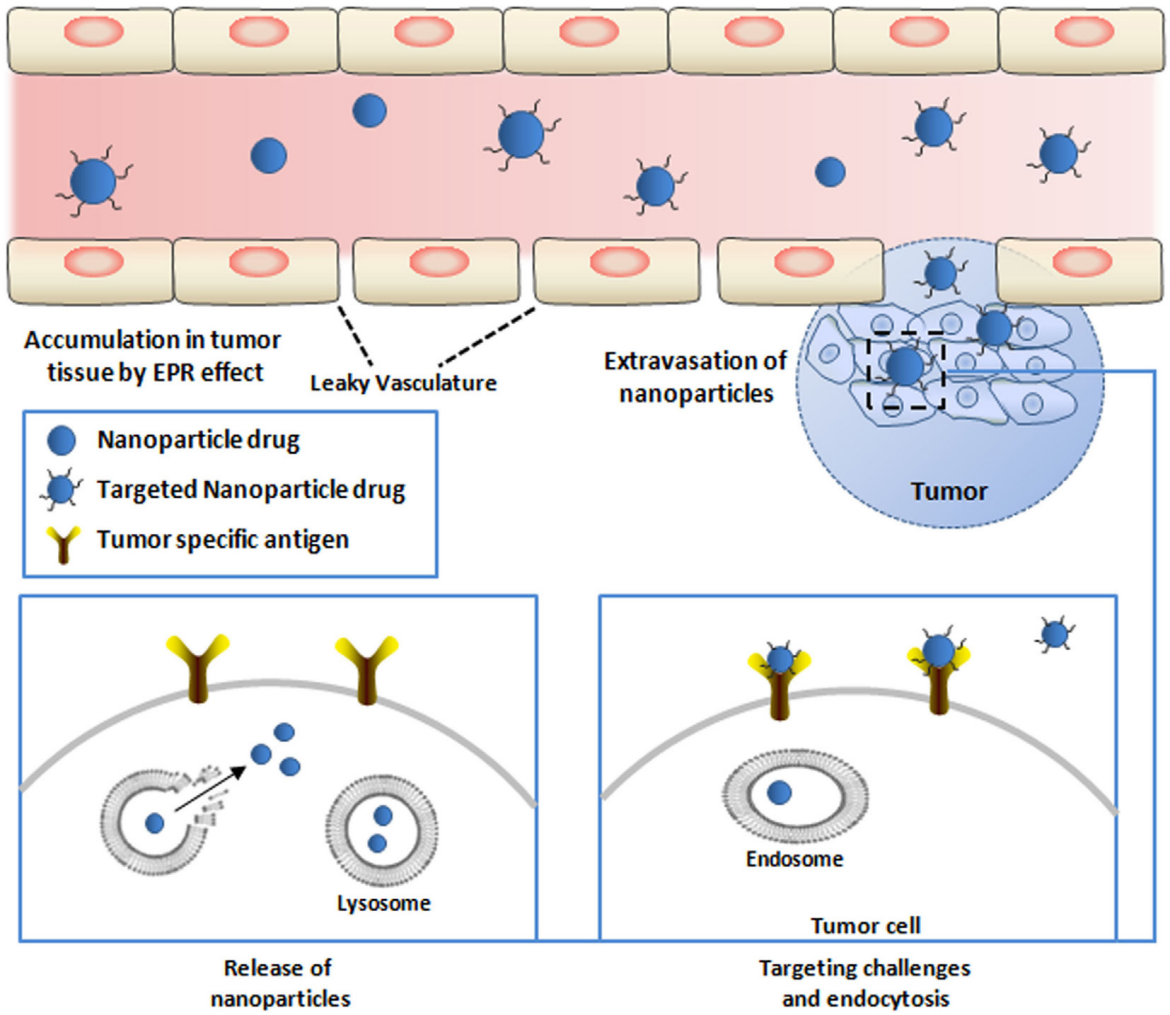
Targeted delivery of therapeutic nanoparticles (NPs) to tumor cells. NPs conjugated with nanobodies (Nbs) against tumor-specific targets are injected into the bloodstream. Circulating NPs need to cross the vascular endothelium of the tumor tissue to infiltrate the tumor site. The endothelium of tumors is poorly formed and allows passage of NPs [causing the enhanced permeation–retention (EPR) effect]. NPs that escape the blood vessel still need to diffuse through the dense extracellular matrix to reach relevant target cells embedded deeply within the tissue. Upon arriving at the surface and attachment with the receptor on the surface of the tumor cells, NPs will be internalized *via* endocytosis (lower right). NPs that are internalized by the cells are conveyed within endosomes, and the release of the active drugs from endosome will exert the antitumor effect (lower left).

The conjugation of drug cargo’s to targeting moieties, especially those against receptors that mediate cellular internalization, was introduced to facilitate the transport of drugs or functional agents in target cells and tissues (Figure 3) (8, 80, 81). Poly-ethylene glycol (PEG) molecules or surface-charge-shielded NPs have been conjugated on the surface of NPs to extend the circulation in the bloodstream leading to more significant accumulation at tumor sites and reduced liver uptake (82–84). The PEG-ylation of NPs also provides chemical reactive moieties to attach bio-functional molecules for specific cell or organ targeting (85, 86). The damaged vasculature around tumor cells will encourage the enhanced permeability and retention (EPR) effect and enhance the accumulation of NPs in the tumor vasculature (Figure 3) (87). After extravasation of NPs into the tumor microenvironment, the interaction between NPs and tumor cells can be enhanced by targeting moieties. Nbs have been employed to serve as targeting molecules, and the delivery systems based on Nbs will be reviewed in the following sections.

#### Nbs Conjugated to Liposomes

Since the discovery of liposome by Bangham in the 1960s, liposomes have been considered as a valuable drug-carrier system, with a morphology and characteristics very similar to those of cellular membranes (88, 89). Liposomes can be constructed in a broad size range from 100 to 400 nm, which might be useful in view of the importance of size in tumor targeting. Significant progress was made over the years and several candidates are at the stage of preclinical evaluation or clinical application (90). The external chemical differences of liposomes can facilitate the construction of targeted systems with Nbs or any other protein and will ultimately result in the accumulation of encapsulated liposomes to tumor tissues (89, 91). In conclusion, Nb–liposome systems play a suitable role for a combined therapeutic strategy and have the potential to enhance the antitumor effect.

##### Anti-EGFR Nbs Conjugated to Empty Liposomes

Oliveira et al. (72) introduced a multivalent Nb–liposome platform without incorporated drugs to target tumors expressing epidermal growth factor receptor (EGFR). Nb EGa1 directed against EGFR was conjugated to the surface of liposomes *via* PEG-ylation. It was shown that the EGa1 C-end modification did not compromise the antigen recognition capacity or change the effective epitope targeting. However, a lower affinity was observed, probably caused by steric hindrance from the attached liposome particles. Nevertheless, a remarkable reduction of more than 90% of cell surface EGFR was observed. Increase of EGa1 on liposomes from 0.4 to 0.8 nmol can even lower the EGFR levels and further enhances the inhibition of tumor growth. The total EGFR protein level in tumors was checked at the end of the study and revealed a remarkable drop in the treated group. Supposedly, the combination of EGa1 to liposomes will retard the clearance from circulation, and the presence of EGa1 ensures specific target interactions, resulting in a measurable increase of accumulated particles in tumor tissues. The poor inhibition of tumor growth *in vivo* is attributed to “blank” liposomes without any drugs incorporated (72).

##### Anti-EGFR Nbs Conjugated to Liposome with Anti-Insulin-Like Growth Factor 1 Receptor (IGF-1R) Kinase Inhibitor (AG538) Encapsulated

In 2012, an improved version of the EGa1-liposome system was introduced by loading the liposomes with an anti-IGF-1R kinase inhibitor (AG538) (Figure 2) (73). IGF-1R plays a crucial role during the progression of particular tumors. It was demonstrated that EGFR inhibition will raise the IGF-1R levels in tumors. Hence, a combined therapeutic strategy against EGFR and IGF-1R was chosen as starting point for this targeted system. The 14C human head and neck cancer cell line and the human epidermoid carcinoma cell line A431 were used to evaluate the tumor inhibition efficacy *in vitro*. Compared to empty EGa1-liposome, a stronger growth inhibition is observed from EGa1-AG538-liposome on 14C cells and empty EGa1-liposomes could decrease the total number of cells by about 40%. This percentage can be increased to nearly 80 by exposure to EGa1-liposome (2 mM TL) with a high dose of free AG538 (80–160 μM) (73). However, the EGa1-AG538-liposomes seemed to be a more robust approach as it reached almost the same inhibition efficacy with just 0.25 mM TL, corresponding to 20 μM AG538. The A431 cell line responded similarly as the 14C cell line. These results strongly support the effective delivery of AG538 by this Nb–liposome system. Next, the *in vivo* antitumor efficacy of EGa1-AG538-liposomes was evaluated in a xenograft model of 14C and MB-468. A strong inhibitory response was observed in the group treated with EGa1-AG538-liposomes, confirming the result of the *in vitro* tests (92). Overall, this kind of platform will encourage the study on different combinations of antitumor drug encapsulated systems and specific antibodies.

##### Anti-EGFR Nbs Conjugated to Extracellular Vesicles (EVs)

Recently, Kooijmans et al. (93) introduced a potent delivery system based on EVs. In this strategy, anti-EGFR Nbs were expressed on the surface of EV fused by glycosylphosphatidylinositol (GPI) anchor signal peptides derived from the decay-accelerating factor. It is demonstrated that the GPI-linked Nbs were successfully displayed and accumulated strongly on the surface of EV. As a result, the targeting efficacy of EVs was dramatically improved *via* their anti-EGFR Nbs, under static conditions. The cancer cell recognition and association was also demonstrated under flow conditions, highlighting the potential of the GPI-anchoring approach and GPI-anchoring drug delivery systems.

##### Nbs Conjugated to Microbubbles

The development of Nb-microbubble (μB) conjugates as a novel molecular tracer has been reported (94). The biotinylated anti-eGFP (cAbGFP4) and anti-VCAM-1 (cAbVCAM1-5) Nbs were site specifically coupled to lipid μBs containing streptavidin. The specific binding of eGFP to μB-cAbGFP4 was confirmed by fluorescent microscopy, as well as the ability of μB-cAbVCAM1-5 to bind VCAM-1 in fast flow. The application of VCAM-1 conjugated μBs as novel molecular ultrasound contrast agent was demonstrated both *in vitro* and *in vivo* (94). It was further proposed that the encapsulation of specific agents in μBs might be used to control a slow release at the tumor site.

#### Nb Conjugation to Micelles

A micelle is an aggregate of amphiphilic block molecules dispersed in aqueous solution with the hydrophilic head regions in contact with the surrounding solvent, sequestering the hydrophobic single-tail regions in the micelle center, ranging from 10 to 100 nm based on the composition and concentration. Micelles are commonly used as platform to deliver hydrophobic drugs, which are difficult to carry through the bloodstream. Micelles remain stable upon dilution and assist in the solubility of these hydrophobic drugs. Their nanoscale dimensions permit an efficient accumulation in tumor tissues *via* the EPR effect (95). For an optimal EPR effect, a long circulation time of drugs or particles is necessary, which might be obtained by coating the small drugs with PEG or to attach drugs to the surface of carriers. Thus, according to the last strategy, the coupling of a targeting moiety (e.g., antibody, scFv, or Nb) to the surface of micelles will increase the accumulation of carriers in target tissue and promote uptake of the specific drugs. Coupling of specific Nbs to the micelle surface will generate a targeted drug delivery system promoting the internalization of carried drugs (71).

##### Anti-EGFR Nbs Conjugated to Micelles

In 2011, a new kind of Nb-micelle drug delivery system was introduced (71). The actively targeted polymeric micelle comprised 80% mPEG-b-p (HPMAm-Lacn) and 20% PDP–PEG-b-p (HPMAm-Lacn) block copolymers. This micelle was decorated with the EGFR antagonist Nb, EGa1, captured to the micellar surface through a disulfide linker (96). The A431 and 14C cell lines and a low endogenous EGFR expressing NIH 3T3 cell line were selected to assess the binding characteristics and uptake of Nb-conjugated micelles. The particles could target effectively to EGFR-positive cells, and no binding was observed to EGFR-negative cells. The results demonstrated that the coupling of EGa1 to the surface of micelles enhanced the recognition of, and uptake by, EGFR-positive target cells.

##### Anti-EGFR Nbs Conjugated to Micelles with Encapsulated DOX

In a follow-up study, an upgraded version (EGa1-DOX-micelle) of this delivery system with encapsulated DOX was proposed (97). Polymeric micelles without Nbs were developed with covalently entrapped DOX through a pH-sensitive linker (Figure 2) (98). Such DOX-micelles showed an increased cytostatic activity against ovarian carcinoma and B16F10 melanoma cells compared to pure DOX. Likewise, in comparison to the free drug, the DOX-micelles exhibited an increased therapeutic efficacy in B16F10 melanoma bearing nude mice. More importantly, mice treated with DOX-micelles showed a prolonged survival compared to the group that received free DOX. The inhibition efficacy to tumor growth dramatically improved by coupling anti-EGFR EGa1 Nbs to the surface of these micelles. EGa1-DOX-micelles were more toxic than the untargeted polymeric micelles for cell lines and xenografted tumors. Early 2010, another biological cargo system was introduced, with EGFR-positive Nbs (EGa1) conjugated to PEG-ylated micelles (98). The subsequent investigation with this delivery system highlighted the importance of the post-insertion strategy, which should target microvesicles to cell lines of interest.

#### Nbs Conjugated to Polymer NPs

Polymer NPs have attracted the interest and have been exploited in different fields over the past decade. This trend originates from their versatile capacities to meet the demands in various applications and marketing requirements. Several types of NPs, including dendrimers, nanospheres, and nanocapsules, have been exploited for enhanced cancer therapy (99–101).

##### Dendrimers

Dendrimers are monodisperse, branched structures, with a size ranging from 3 to 20 nm (102). The surface of dendrimers can be functionalized by coupling targeting moieties. Functional agents can be encapsulated in the dendrimer’s multifunctional core to facilitate drug delivery. Drug molecules, such as paclitaxel, can also be attached to the exterior of the dendrimer for special purposes. Recently, DOX was conjugated to carboxyl-terminated poly(amidoamine) dendrimers (PAMAM) and assessed against lung metastases for improved pharmacokinetics and biodistribution (103). A dramatic increase in efficacy of DOX treatment was observed, upon pulmonary administration, in a lung metastasis mouse model bearing the B16-F10 melanoma. A decreased tumor weight and increased survival rates of the animals (C57BL/6) were noted. Compared to free DOX, this conjugate was demonstrated to further increase the therapeutic efficacy as indicated by the fewer number of nodules observed in lungs. The results demonstrated that pulmonary administration of DOX conjugated to PAMAM dendrimer is a useful strategy to enhance the therapeutic efficacy and decrease systemic toxicity of DOX. The conjugation of specific Nbs to the surface of dendrimer is expected to further facilitate tumor targeting (Figure 2).

##### Nanospheres

A nanosphere is a delivery vehicle composed of a spherical polymeric matrix ranging from 1 to 100 nm, where the drug can be encapsulated inside the aqueous or oily core from where it is released slowly during the circulation in the bloodstream. The surface of the nanosphere can also be PEG-ylated to increase the half-life and to facilitate the binding of Nbs for targeted therapy (Figure 2) (104, 105).

##### Nanocapsules

Nanocapsules are nanoscale shells of 10–1,000 nm with drugs encapsulated inside their core and separated from the environment by a polymeric membrane (106, 107). Nanocapsules are used in a myriad of fields, including medical applications for drug delivery, food enhancement, nutraceuticals, and self-healing materials. The most attractive current application is the targeted delivery of agents to particular tissues. Monomer, bivalent, or even trivalent Nbs can be attached to the surface of this delivery system to obtain a specific targeting (Figure 2) (108). Although there is no publication yet where such multimeric Nbs are conjugated on nanocapsules, it remains a very attractive material of high potential for future research.

##### Nbs Conjugated to Albumin NPs

Another type of a highly potent delivery module comprises albumin NPs. Albumin is the most abundant plasma protein in the bloodstream, participating in several important regulations. Moreover, albumin is biocompatible and bio-safe, and albumin NPs as a drug delivery system was proposed by Muller et al. (109). The work inspired several researchers to develop such albumin NPs into a safe drug delivery system (110, 111). In one of these publications, a novel albumin nanoparticle drug carrier system (NANAPs) was loaded with the multi-kinase inhibitor 17864. Furthermore, their anti-EGFR Nb EGa1 was linked *via* maleimide functionalized PEGs and coated to the surface of these NPs to reinforce the target delivery to EGFR-positive 14C squamous head and neck cancer cells (Figure 2) (111). PEG-ylated NPs without EGa1 on their surface showed lower targeting and internalization efficacy compared to PEG-NP-EGa1. After binding to the cancer cells, the clathrin-mediated endocytosis leads eventually to the lysosomal degradation of the NPs releasing the multi-kinase inhibitor 17864 inside cells and provoking a notable anti-proliferative effect on tumor cells. The importance of a targeted effect from the EGa1 Nb on NANAPs was demonstrated *in vitro*, whereas the cell proliferation inhibition was not observed by treating cancer cells with non-targeted NPs encapsulated with 17864.

### Targeted Therapy with Nb-Decorated Viral Vectors

Gene therapy with the assistance of viral vectors has become a very important technology in basic life sciences and applied medicine. To date, viruses, such as adenovirus, adeno-associated virus (AAV), and herpes simplex virus, are favored for this task (112, 113). LV and AAV are well-established vectors (114, 115), and these viruses can be employed to transfer genes, including those encoding Nbs into the host cells to produce intracellular Nbs (i.e., intrabodies). The LV is the most studied model for gene delivery and immunotherapy. Unfortunately, it remains challenging to deliver the genes of interest within the lentiviral particles after an *in vivo* administration to relevant target cells such as tumor cells or antigen-presenting cells (APCs) (116). The administration of wild-type AAV and LV vectors usually results in virion accumulation in liver and spleen.

**Figure 4:**
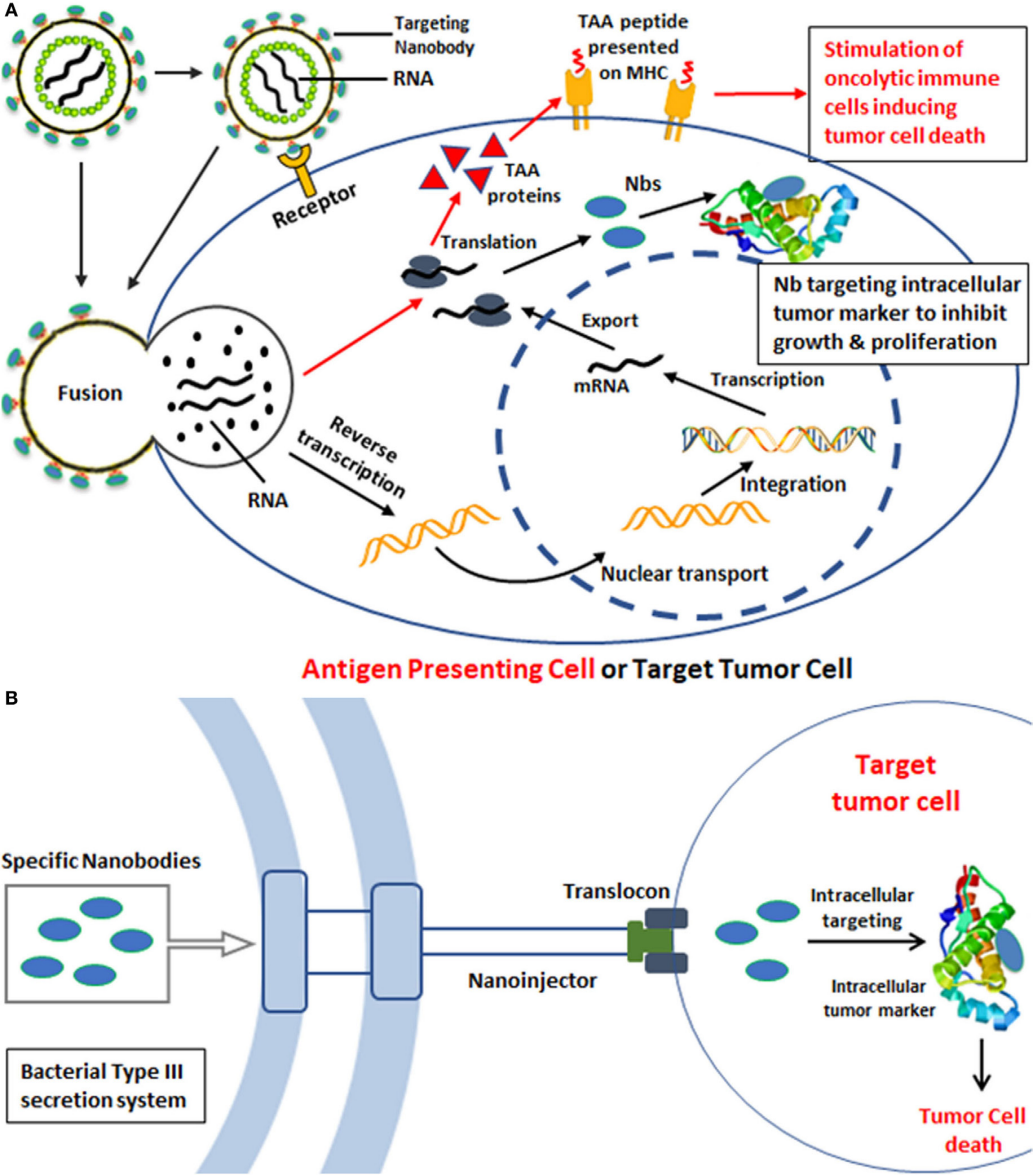
Strategies for intracellular tumor targeting. **(A)** Gene delivery of specific nanoboides (Nbs) against intracellular tumor targets based on lentiviral vectors. The lentivirus displays Nbs directed toward antigen-presenting cells (APCs) such as dendritic cells (DCs). The initial attachment of the virus to the cellular receptor on the surface of APC allows internalization of the viral contents. The viral nucleoprotein core containing the genomic RNA is released into the cytoplasm after entry. Reverse transcription and synthesis of full-length chimeric viral DNA produces an integration-competent nucleoprotein complex that mediates integration of viral DNA into the host cell genome. Integrated chimeric viral DNA serves as a transcription template for the synthesis of tumor-associated antigens that after proteolysis will be presented on MHC to stimulate oncolytic T cells inducing tumor cell death (red arrows). In an alternative approach, the LVs could contain genes encoding Nbs against intracellular tumor markers. The targeting of such LVs to tumor cells could then produce intrabodies (Nbs) that will associate with the intracellular tumor marker to inhibit tumor growth and proliferation (black arrows). **(B)** Transport of specific Nbs into tumor cells *via* bacterial type III secretion system (T3S) for intracellular tumor targeting. Gram-negative bacteria use a specialized secretion apparatus known as the T3S system to inject proteins directly into the eukaryotic cells, such as *Y. enterocolitica* T3S, *S. typhimurium* T3S, and *E. coli* T3S. Bacterial proteins that are delivered by a T3S are injected through the eukaryotic cell membrane *via* a proteinaceous transmembrane channel known as the type III translocon. The schematic components of the T3S nanosyringe are shown and Gram-negative bacteria were engineered to produce antigen-specific Nbs against intracellular tumor markers. The attachment of T3S and tumor cells will facilitate the export of Nb proteins inside tumor cells, such as HeLa cells. These internalized Nbs will block and inhibit the signaling cascades or processes of tumor metastasis, leading to targeted cancer therapy.

Breckpot et al. (116) developed an interesting approach, whereby a modified LV vector was assembled with a binding-defective, but fusion competent, envelope glycoprotein derived from VSV-G decorated with Nb DC2.1 (Figure 4A). This Nb targets dendritic cells (DCs) that together with macrophages are imperative for activation of antigen-specific T cells. Such APCs are often targeted in immunotherapeutic strategies for the treatment of cancer and infectious diseases (117, 118). The modified LVs contain genes for tumor-associated antigens (TAAs) (119). Upon transduction of the APC, the TAA will be processed and presented to oncolytic effector cells that will subsequently eradicate cancer cells, both primary and metastasized.

The transducing specificity of Nb DC2.1 displaying LVs was evaluated on both, mouse and human APCs. It was demonstrated that DC2.1 Nb-displaying LVs showed an Nb-dependent and APC-specific transduction on murine cell lines and *in vitro* generated DCs (116). *In vivo* transduction with the DC2.1 Nb-displaying LVs was demonstrated after intranodal injection by bioluminescence imaging, and the transduction results were confirmed by nested PCR (116). Phenotypic characterization of the *in situ* transduced lymph node (LN)-derived DCs demonstrated that the entry of DC2.1 Nb-displaying LVs showed a tendency to accumulate at macrophages, conventional DCs (cDCs), and plasmacytoid DCs. Importantly, myeloid DCs are supposed to mediate immune responses upon LV transduction, and the transduction was enhanced with Nb-displaying LVs.

The use of Nbs on LVs (such as R3_13 LVs) can target specifically to human LN-resident myeloid DCs (120). Later on, an extended study was performed to check the targeting of LVs to cDCs, which are assumed to play a central role in the induction of a TH1-mediated antitumor immune response (117). In this study, the *in vivo* transduction profile and immune stimulatory potential of broad tropism LVs was compared with non-targeting BCII10 Nbs and APC-targeting Nb DC1.8 or DC2.1 displaying LVs. It was demonstrated that the DC1.8-LVs can exclusively transduce cDCs, while also macrophages and pDCs can be transduced with DC2.1-LVs. The transduction of these different cell types opens the potential to stimulate both, the antigen-specific CD8+ and CD4+ T cells (121). Intranodal immunization with ovalbumin encoding LVs induces the proliferation of antigen-specific CD4+ T cells. It has been demonstrated that all targeted LVs were able to stimulate cytotoxic T lymphocytes, and the APC-targeted LVs were as potent in therapy as broad tropism LVs and as such meet their expectations as safer and efficacious LV-based vaccines (117).

## Intracellular Targeting of Tumors with Nbs

To date, the majority of the research focuses on extracellular tumor markers, including cytokines, signaling receptors, and extracellular domains of cell surface proteins (122). However, most of the signaling regulation linked to the growth and proliferation of tumor cells occurs intracellularly, and thus intracellular markers should be promising therapeutic targets as well (123). The barrier of the lipid membrane halts the transfer of Nbs to the intracellular compartment, but many researchers try to develop potent delivery strategies to transport intact or at least functional Nbs into cells, and several strategies have been explored for their intracellular transportation. The LVs can be engineered to target to tumor cells *via* decoration with tumor surface marker-specific Nbs, while encoded Nbs within the LVs might produce intracellular Nbs that could associate with intracellular tumor markers to inhibit growth and proliferation (Figure 4A).

Alternatively, bacteria have developed sophisticated systems, such as the type III secretion system (T3SS), to translocate exogenous proteins into eukaryotic host cells (124). This needle-like system serves as a sensory probe to detect eukaryotic organisms and to inject bacterial proteins directly in the host cell to prepare these cells and to assist the pathogen to survive and to escape the host immune system.

### *E. coli* Bacteria T3SS

An old observation, that bacteria are homing at the hypoxic environment of solid cancers, inspired researchers to harness bacteria with Nbs that would be translocated *via* the T3SS inside the cancer cells to cause damage (Figure 4B). In a first step to explore this strategy, it was demonstrated that the non-invasive *E. coli* bacteria carrying a T3SS could translocate successfully the Nbs into mammalian cells (125). Several constructs of Nb fused to EspF_20_ T3 signal (T3s) were cloned into non-pathogenic *E. coli* cells and shown by western blot to get inside HeLa cells. The immunoprecipitation further demonstrated the intact antigen-binding activity of the injected Nbs (125). Higher levels of injected T3s-Nbs remained inside the HeLa host when they express the cognate antigen. Thus, the non-invasive bacterial T3SS is a promising strategy to deliver Nbs into mammalian cells to target intracellular cell components and signaling pathway molecules.

### The *Y. enterocolitica* T3SS

Likewise, Ittig et al. (126) reported a protein delivery system based on the type III secretion of *Y. enterocolitica*. In their research, YopE, a *Y. enterocolitica* effector with Rho GTPase-activating protein activity, was utilized for the expression of protein–YopE fusions in *Y. enterocolitica* and translocation of proteins into mammalian cells. The secretion of multiple proteins was evaluated, including cell-cycle proteins (i.e., Mad2, CDK1, CDKN2A/INK4A, CDKN2B/INK4B, and CDKN2C/INK4C), apoptosis-related proteins(Bad, FADD, caspase-3 [CASP] p17 and p12, zebrafish BID, and zebrafish t-BID), and signaling proteins (TRAF6, TIFA, and the GPCR Gα subunit GNA12). Furthermore, an anti-GFP Nb (VHH GFP4) and a VHH GFP4 fusion construct for targeted protein degradation was also tested (126). The delivery of these functional fusion Nb proteins of different size and structure was demonstrated after infection of HeLa cells. The Nb against GFP was employed to assess the translocation of functional Nbs from *Y. enterocolitica* into HeLa cells. Interestingly, the translocation of YopE1-Nb fusions occurs first in the cytoplasm, whereas translocated YopE1-Nb fusions against GFP were exclusively detected in the nucleus of cells expressing histone 2B-GFP, illustrating the mobility of the fusion inside the cell and its organelles and the interaction between the Nbs and their target antigen (126). The results indicated that YopE fusion are effectively secreted and delivered into eukaryotic cells and that the *Y. enterocolitica*-based delivery is fast, homogenous, and controllable. However, the unspecific targeting of bacteria to normal and tumor cells indicates that further engineering of the bacteria will be required to obtain an exclusive tumor-specific Nb translocation. This might be achieved by anchoring tumor-specific Nbs to the surface of the engineered bacteria.

### *Clostridium*-Directed Antibody Therapy

A non-toxic bacteriolytic strain of *Clostridium* has been engineered for the production of tumor therapeutic proteins (127). This obligatory anaerobic *Clostridium* specifically colonizes hypoxic and necrotic regions present in solid tumors but normally absent in other parts of the body. The efficacy of *Clostridium*-directed tumor therapy (CDAT) has been demonstrated in experimental models as a vehicle for tumor-specific delivery of prodrug converting enzymes (128, 129) and to enhance radiotherapy and chemotherapy (130–132).

Nanobodies targeting HIF-1 were cloned in *Clostridium novyi-NT* and *C. sporogenes* strains. The expression of HIF-specific intrabodies in an oncolytic *C. novyi* strain opened the path for developing a *Clostridium*-directed antibody therapy (133).

## Molecular Imaging with Nbs for Early Stage Diagnosis of Tumors

Early diagnosis is essential to increase chances on a successful treatment of tumors. Recently, Nbs supported by their small size, high stability, and high target specificity and affinity have been engineered into Nb-detective constructs for non-invasive *in vivo* molecular imaging (33). These molecules reach rapidly a maximal contrast between signal in the pathological tissues and that in healthy tissues, which is crucial for optimal *in vivo* molecular imaging. The short half-life of Nbs in the bloodstream due to rapid clearance of excess of non-targeting Nbs *via* kidney and bladder guarantees a high tumor to background ratio at early time points after administering the Nb probe. To date, several imaging techniques have been developed and applied for clinical application, such as positron emission tomography (PET), single photon emission computed tomography (SPECT), computed tomography (CT), magnetic resonance imaging (MRI), optical, ultrasound, and photo-acoustic imaging (134–136). In following sections, radionuclide imaging (by SPECT and PET) will be described as it passed a phase I study (136) and probes based on Nbs developed during the past decade are listed in Table 1.

**Table 1:** Conjugated systems based on nanobodies (Nbs), employed for imaging of tumors or for drug delivery to cancer cells.

Construct	Cellular target of Nb	Effector domain	Cancer cell lines	Reference
Nb-toxin	Carcinoembryonic antigen	*Enterobacter cloacae* β-lactamase fused to Nb	Mice bearing LS174T xenografts	(75)

	Malignant glioblastoma multiforme	Tumor necrosis factor-related apoptosis-inducing ligand (TRAIL) fused to Nb	U87-mCherry-FLuc cells into the brains of nude mice	(76)

Vascular endothelial growth factor receptor-2	*Pseudomonas* exotoxin A (PE38) fused to Nb	HEK293, 293KDR cells	(77)

Nb-polymer NP	Epidermal growth factor receptor (EGFR)	Liposome fused to Nb EGa1	14C human head and neck cancer cell line and the human epidermoid carcinoma cell line A431	(72, 73)
		Nb EGa1-liposome encapsulated AG538 anti-insulin-like growth factor 1 receptor kinase inhibitor (AG538)
		Nb EGa1-extracellular vesicles (EVs) fused to glycosylphosphatidylinositol (GPI) anchor signal peptides derived from decay-accelerating factor	Neuro2A cells, human epidermoid carcinoma cells A431 and HeLa cells	(93)
	
	EGFR	Nb EGa1-micelles	A431, 14C cell line and low endogenous EGFR expression NIH 3T3 cell lines, ovarian carcinoma and B16F10 melanoma cells	(71, 97, 98)
		Nb EGa1-micelles encapsulated doxorubicin (DOX)		
	
	EGFR	Nb EGa1-albumin nanoparticles encapsulated multikinase inhibitor 17864	EGFR-positive head and neck squamous cell carcinoma cell line UM-SCC-14C	(111)
		Dendrimers-encapsulated DOX	Mouse melanoma (B16-F10) and Male C57BL/6 mice	(102, 103)

Nb-lentivirus	Dendritic cells (DCs) and macrophages	Nb DC2.1 decorated lentiviral vectors for specific gene delivery specific targeting	HEK 293T, mouse fibroblasts NIH 3T3 cells, mouse leukemic macrophage RAW264.7 cells, mouse T-lymphoma EL4 cells, and mouse B-lymphoma A20 cells	(116, 117, 137)

Molecular imaging	EGFR	^99m^Tc for single photon emission computed tomography (SPECT) on Nb 8B6	Human epidermoid carcinoma (A431), human prostate carcinoma (DU145)	(44)
		^99m^Tc for SPECT on Nb 7C12	Human epidermoid carcinoma (A431)	(33, 136, 138, 139)
		^99m^Tc for SPECT on Nb 7D12 ^68^Ga for PET on Nb 7D12 IRDye800CW for optical imaging on Nb 7D12		

	Human epidermal growth factor receptor-2 (HER-2)	^99m^Tc for SPECT on Nb 2Rs15d	Human colon carcinoma (LS174T), human breast cancer (SKBR3), and human ovarian cancer (SKOV3)	(139, 140)
		^68^Ga for PET on Nb 2Rs15d	Human ovarian cancer (SKOV3)	
		IRDye800CW for optical imaging on Nb 11A4	Human breast cancer (SKBR3)	(55)

	Vascular cell adhesion protein 1 (VCAM1)	^99m^Tc for SPECT on Nb VCAM1-5	Atherosclerosis (ApoE-deficient mice)	(94, 141)
		Microbubble for ultrasound imaging onto Nbs	Murine adenocarcinoma (MC38)	

Intracellular delivery of specific Nbs	Amylase (Amy) and the green fluorescent protein	Nb Vamy and Vgfp-EspF20 T3 signal (T3s)	HeLa CCL-2, HEK 293T, and Swiss 3T3 fibroblasts	(125, 126)
		Nb against EGFP-YopE based on type III secretion system		

	HIF-1α	Intrabodies produced by *Clostridium novyi*-NT strain	–	(133)

### Nbs in Nuclear Imaging

For nuclear imaging, different radionuclides (e.g., ^99m^Tc, ^89^Zr, ^68^Ga, ^18^F, or ^64^Cu) are employed for labeling target-specific Nbs. Huang et al. (44) adapted the labeling with ^99m^Tc-tricarbonyl intermediate [^99m^Tc(H_2_O)_3_(CO)_3_] for an anti-EGFR Nb (8B6) and used this probe for non-invasive imaging with SPECT. A rapid blood clearance (half-life ~1.5 h) of such conjugates was demonstrated and the potential to differentiate tumors with high or low levels of EGFR (44, 142). Likewise, Gainkam et al. (45) performed a similar experiment with two different Nbs using pinhole SPECT and micro-CT. The same group evaluated the relationship between tumor uptake of the EGFR-specific Nb ^99m^Tc-7C12 and the tumor burden, as well as the possibility to monitor tumor response to erlotinib with this probe (143). A good correlation between tumor uptake of ^99m^Tc-7C12 with tumor burden was observed. Thus, ^99m^Tc-7C12 seems to be a promising tool to monitor the therapeutic response and treatment progress in EGFR overexpressing tumors (138).

Besides EGFR, the human epidermal growth factor receptor type 2 (HER2) is also an interesting target for molecular imaging, as one-quarter of all breast cancers is overexpressing HER2 (136). This biomarker is also the target for Trastuzumab. For stratification, it would be preferable to screen breast cancer patients by non-invasive *in vivo* imaging for occurrence of HER2 on their tumors. To this end, multiple Nbs were evaluated to identify a lead Nb (2Rs15d) for imaging of HER2-positive tumors that does not interfere with the binding of the therapeutic Trastuzumab for the same target (140). The accumulation on the tumor tissue was demonstrated by *in vitro, ex vivo*, and *in vivo* assays. The *in vivo* assay in mice bearing HER2-expressing tumor xenografts confirmed the high uptake in tumor tissue, with low level of detection at healthy tissues (except for kidneys). Later on, it was shown that the removal of hemaglutinin tag and the His tag on the Nb decreased the kidney retention of the probe drastically (139, 140).

The reduced radio-toxicity at kidneys with “tag-stripped” Nbs allows a switch to targeted radionuclide therapy (TRNT) by changing the label on Nb 2Rs15d to ^177^Lu (139). Although TRNT has been a promising strategy for tumor killing, the undesirable pharmacokinetics (prolonged serum retention and poor tumor penetration) of mAb vehicles carrying the radiotoxic load curtailed the application. Therefore, the substitution of mAbs by Nbs having favorable pharmacokinetics and a highly specific target accumulation leads to a low accumulation of label in healthy tissues. Indeed, it has been demonstrated that Nb-based TRNT could target tumors successfully in a xenograft model. This highlights the potential of Nb-based TRNT as a valuable candidate for tumor diagnosis and therapy (144).

### Nbs in Optical Imaging

Various methods allow optical molecular imaging where contrast is obtained by fluorescence, bioluminescence, absorption, or reflectance. The most valuable features of optical imaging compared to other imaging techniques include the high safety, high flexibility of the probes, and high sensitivity for the targets (135, 145, 146).

In 2012, a novel platform for optical imaging with Nbs was developed whereby the anti-EGFR Nb 7D12 and Cetuximab were conjugated with the near-infrared (NIR) fluorophore IRDye800CW (33). The 7D12-IR allowed the visualization of tumors as early as 30 min post-injection of the probe, whereas Cetuximab-IR failed to provide a signal at the tumor site above background. Hence, the anti-EGFR Nb conjugated to the NIR fluorophore was demonstrated to possess excellent properties, which will facilitate preclinical or clinical optical imaging applications.

In 2016, Kijanka et al. (147) reported a combination of optical conjugations based on two different Nbs against two different breast tumor markers, for an improved tumor detection: Nb B9 against CAIX, which targets the peri-necrotic regions of tumors, and Nb 11A4 against HER2. This dual-spectral imaging strategy accomplishes successfully the optical molecular imaging of CAIX and HER2-positive DCIS xenografts *in vivo*, under conditions mimicking surgical settings. This strategy is assumed to facilitate a faster detection of tumor markers, and it is highly promising to utilize the dual-spectral imaging strategy for the early diagnosis, treatment program planning, and monitoring the treatment response (147).

### Nb Imaging Based on Ultrasound

Ultrasound imaging is widely used for medical applications by collecting sound waves reflected by tissues and organs. Microbubbles (μBs) have been developed as ultrasound contrast agents and can be targeted to tumors by conjugation with specific Nbs. Specific Nbs (μB-cAbVCAM1-5) against the vascular cell adhesion protein 1 (VCAM1) were introduced for μBs targeting (94, 141). The enhancement of ultrasound imaging was observed both *in vitro* and *in vivo*. Although the detection and imaging of this technique has been restricted to the systemic vasculature, it is still worth extending the effort toward other targets (148).

## Conclusion and Future Perspectives

Soon after the initial report on functional heavy chain antibodies in camelidae, Nbs have been introduced in different areas, such as in oncology as antagonists, for the development of Nb-conjugated drug delivery systems, and for molecular imaging. This report contains an overview of drug delivery systems using Nbs, including transport of specific agents to extracellular tumor targets and highly potential intracellular tumor markers. The cellular imaging techniques based on Nbs were also summarized to provide basic knowledge and promising insights from further clinical application. It is well established that antagonists of small size need to exhibit a high affinity and specificity for their cognate target, so that they can associate to the target before being cleared *via* the kidneys. Nbs have demonstrated to fulfill this task. Generally, Nbs seem to be very promising when used as targeting moieties to develop novel drug delivery systems or to generate an intracellular targeting agent (149).

Several different strategies have been developed to broaden the application range of Nbs for diagnosis and cancer therapy: in first instance “naked” Nbs are utilized, in bivalent or bispecific formats to act as antagonists against tumor angiogenesis, metabolism, and metastasis. The small size of Nbs facilitates extravasation and solid tumor penetration. A further development consists in conjugating Nbs with toxins to create a specific drug delivery system. This toxic agent can be conjugated directly to Nbs or can be anchored onto or within NPs, consisting of liposomes, micelles, or polymers. The size and format of these drug delivery systems is crucial and greatly affects its accumulation at the tumor site. Systems that increase the size of the Nb enhances concomitantly its retention in blood circulation and conversely, decreases tumor penetration.

To date, most of the applications just employed Nbs directed against extracellular targets. However, intracellular effectors (e.g., components of signaling cascades) are thought to be excellent therapeutic targets for Nbs as well. In this case, the plasma membrane will block the transport of Nb-based inhibitors into cells, obviously restricting the application. Potential strategies to transfer Nbs inside cells include LVs harnessed with Nbs for cellular targeting or Nbs delivered *via* the bacterial T3 secretion system. Trials with LVs revealed the potential to target different cell types. The simplicity to engineer Nbs permits the recognition of any cell type and subsequent display technology will further enhance the potential of LVs for gene therapy purposes, tumor immunotherapy, and intracellular targeting. Alternatively, protocols to employ T3S have been developed to inject heterologous type III and IV effectors (150, 151), as well as mammalian proteins inside cells. This T3S-based protein delivery strategy could facilitate the transfer of particular antagonist into cells and induce apoptosis (126). It was suggested that transport of Nbs into cells by non-pathogenic bacterial strains equipped with T3S would be a promising technology to target host cells and intracellular signaling pathways.

While *in vivo* molecular imaging with Nbs is mainly relying on SPECT or PET, alternative innovative techniques, such as optical and ultrasound imaging, are being developed (134, 136). Since all imaging technology has its weakness, it is probably best to combine multiple techniques to exploit synergistic advantages and multimodal contrast agents or imaging probes score high on the wish list.

Obviously, Nbs are a versatile tool that will fulfill a central role in various clinically relevant applications.

## Author Contributions

All authors listed have made a substantial, direct, and intellectual contribution to the work and approved it for publication.

## Conflict of Interest Statement

The authors declare that the research was conducted in the absence of any commercial or financial relationships that could be construed as a potential conflict of interest.
